# Low-dose naltrexone’s utility for non-cancer centralized pain conditions: a scoping review

**DOI:** 10.1093/pm/pnad074

**Published:** 2023-06-11

**Authors:** Adam Rupp, Erin Young, Andrea L Chadwick

**Affiliations:** Department of Physical Medicine and Rehabilitation, University of Kansas Medical Center, Kansas City, KS 66160, United States; Department of Anesthesiology, University of Kansas Medical Center, Kansas City, KS 66160, United States; Department of Anesthesiology, University of Kansas Medical Center, Kansas City, KS 66160, United States

**Keywords:** low-dose naltrexone, chronic pain, centralized pain, Crohn’s disease, inflammatory bowel disease, complex regional pain syndrome, fibromyalgia, diabetic neuropathy, rheumatoid arthritis, low back pain, glial cells, inflammatory modulation

## Abstract

**Background:**

At low doses, naltrexone (LDN) has been shown to modulate inflammation through the interruption of microglial cell activation within the central nervous system. One of the most likely contributors to centralized pain is changes in microglial cell processing. Therefore, it has been postulated that LDN can be used to manage patients with pain resulting from central sensitization due to this relationship. This scoping review aims to synthesize the relevant study data for LDN as a novel treatment strategy for various centralized pain conditions.

**Methods:**

A comprehensive literature search was conducted in PubMed, Embase, and Google Scholar, guided by the Scale for Assessment of Narrative Review Articles (SANRA) criteria.

**Results:**

Forty-seven studies related to centralized pain conditions were identified. Many of the studies were case reports/series and narrative reviews, but a few randomized control trials have been conducted. Overall, the body of evidence revealed improvement in patient-reported pain severity and in outcomes related to hyperalgesia, physical function, quality of life, and sleep. Variability in dosing paradigms and the time to patient response was present in the reviewed studies.

**Conclusions:**

Evidence synthesized for this scoping review supports the ongoing use of LDN for the treatment of refractory pain in various centralized chronic pain conditions. Upon review of the currently available published studies, it is apparent that further high-quality, well-powered randomized control trials need to be conducted to establish efficacy, standardization for dosing, and response times. In summary, LDN continues to offer promising results in the management of pain and other distressing symptoms in patients with chronic centralized pain conditions.

## Introduction

Naltrexone is an opioid antagonist that was first developed in 1963.^1^ It has historically been used at higher doses (50–100 mg) to treat opioid and alcohol dependence through its antagonistic action on the µ-opioid receptor.^2^ Full-dose naltrexone’s binding to the µ-opioid receptor blocks the euphoric effect from opioids, thus helping to reduce overall opioid use.^3^ The µ-opioid receptor is in a family of G-protein–coupled receptors that, when activated, modulate presynaptic and postsynaptic calcium channels, attenuating the excitability of neurons.^4^

Low-dose naltrexone (LDN) (4.5 mg), on the other hand, was first described in the 1980s as a treatment option to help with inflammation.^5^ Since that time, the use of LDN has expanded to treat a variety of medical conditions and syndromes, including chronic pain disorders, chronic fatigue, migraine headaches, and certain skin conditions.^1^ The beneficial properties seen at lower doses of naltrexone are due to a paradoxical effect that reduces its affinity for the µ-opioid receptor and instead makes it bind more readily to the toll-like receptor 4 (TLR4).^6^ TLR4 is located on the microglial cells of the central nervous system (CNS), and this receptor family plays a large role in modulating the inflammatory and cytokine systems, whereby activation of TLR4 receptors increases activation of glial cells, which in turn leads to increased proinflammatory cytokine production.^7^ These modulation effects allow LDN to be used for various chronic pain states in which neuroinflammation has been implicated, including but not limited to complex regional pain syndrome (CRPS), fibromyalgia (FM), and irritable bowel syndrome. Initial efficacy and tolerance studies have shown improvements in pain and symptom severity and in functional scores, with minimal side effects.^8^

Chronic pain affects an estimated 11% to 40% of the US population^9^ and is associated with negative patient outcomes, including decreased function, reduced quality of life, and significant socioeconomic impacts.^9^ Although multiple factors have been implicated in the development of chronic pain, central sensitization has been identified as a primary pathophysiological process in most chronic pain syndromes.^10^ The mechanisms underlying the development of central sensitization are complex and multifactorial; they include changes in neuronal response properties and integration of nociceptive information in the CNS.^11^ Of particular relevance to the present study, abnormal pain processing mediated by alterations in microglial structure/function could play a role in perpetuating sensitization, separate from any direct physical damage or inflammation to neurons.^10^ Microglia are resident CNS macrophages and serve a primary defense role in the brain and spinal cord. Normally in a quiescent state, microglia become activated by typical immune triggers such as cell death, peripheral inflammation, and infection. Once activated, they undergo morphological changes and release a broad profile of cytokines and other proinflammatory mediators that are capable of altering response properties in individual neurons and within neuronal networks. Unfortunately, abnormal or repeated activation of microglia can lead to augmented activation and ultimately heightened responses to pain signals, leading to central sensitization.^11^

Although the term “central sensitization” refers to specific changes in CNS neuronal response properties, central sensitization is characterized behaviorally by the presence of hyperalgesia and allodynia, defined as increased pain response to noxious and non-noxious stimuli, respectively.^12^ Centralized pain conditions are strictly a clinical diagnosis of exclusion. Patients usually have >3 months of widespread or multifocal allodynia and hyperalgesia without physical exam, neurological, or laboratory findings that would account for the pain. Often, however, there will be other concurrent symptoms, such as memory loss, fatigue, depression, or anxiety.^13^ Centralized pain syndromes are grouped under the new International Association of the Study of Pain definition of “nociplastic pain,” in which the experience of pain is present in the absence of clear evidence of actual or threatened tissue damage or evidence for disease of the somatosensory system.^14^ As such, with nociception occurring with no clear peripheral or central lesion or pathology, treatment of centralized pain is complex and challenging. Currently, limited treatment options exist for patients with chronic centralized pain. For many of the available options, including behavioral therapy, antidepressants, neuropathic medications, and exercise, the efficacy and longevity are limited.^15^

The goal of the present scoping review is to synthesize the available evidence related to LDN and its utility for various centralized pain conditions, as well as to highlight any knowledge gaps to guide development of well-designed randomized control trials (RCTs) and other research studies leading to evidence-based clinical practice guidelines.

## Methods

With the assistance of a professional librarian, a comprehensive literature review was conducted in PubMed, EMBASE, and Google Scholar, with publication dates ranging from January 1, 2000, to February 1, 2022. Article eligibility and inclusion criteria were limited to publications in the English language and studies pertaining to human subjects. Exclusion criteria included studies focusing on the use of full-dose naltrexone or the use of naltrexone for alcohol and opioid abuse, obesity, dermatological conditions, or other nonpainful disease processes (amyotrophic lateral sclerosis, primary sclerosing cholangitis, Hailey-Hailey disease, opioid-induced constipation, and chronic fatigue). Studies involving cancer- or chemotherapy-induced neuropathy or pain were also excluded. All other studies reporting on patients using LDN for chronic pain were included. Keywords, Medical Subject Headings, and EMTREE subject headings were used to search for the concepts of LDN and chronic pain. Keywords included “low dose naltrexone” and “chronic pain”, “complex regional pain syndrome”, “inflammatory bowel disease”, “fibromyalgia”, “low back pain”, “rheumatoid arthritis”, “neuropathy”, and “migraines”. The numerical results from the literature search are represented in Table 1.

**Table 1.** Number of results based on search terms from PubMed, EMBASE, and Google Scholar

Search term	Results
Low dose naltrexone	723
LDN and chronic pain	82
LDN and complex regional pain syndrome	9
LDN and irritable bowel disease	29
LDN and fibromyalgia	38
LDN and low back pain	8
LDN and rheumatoid arthritis	5
LDN and neuropathy	11

LDN = low-dose naltrexone.

**Figure 1.**
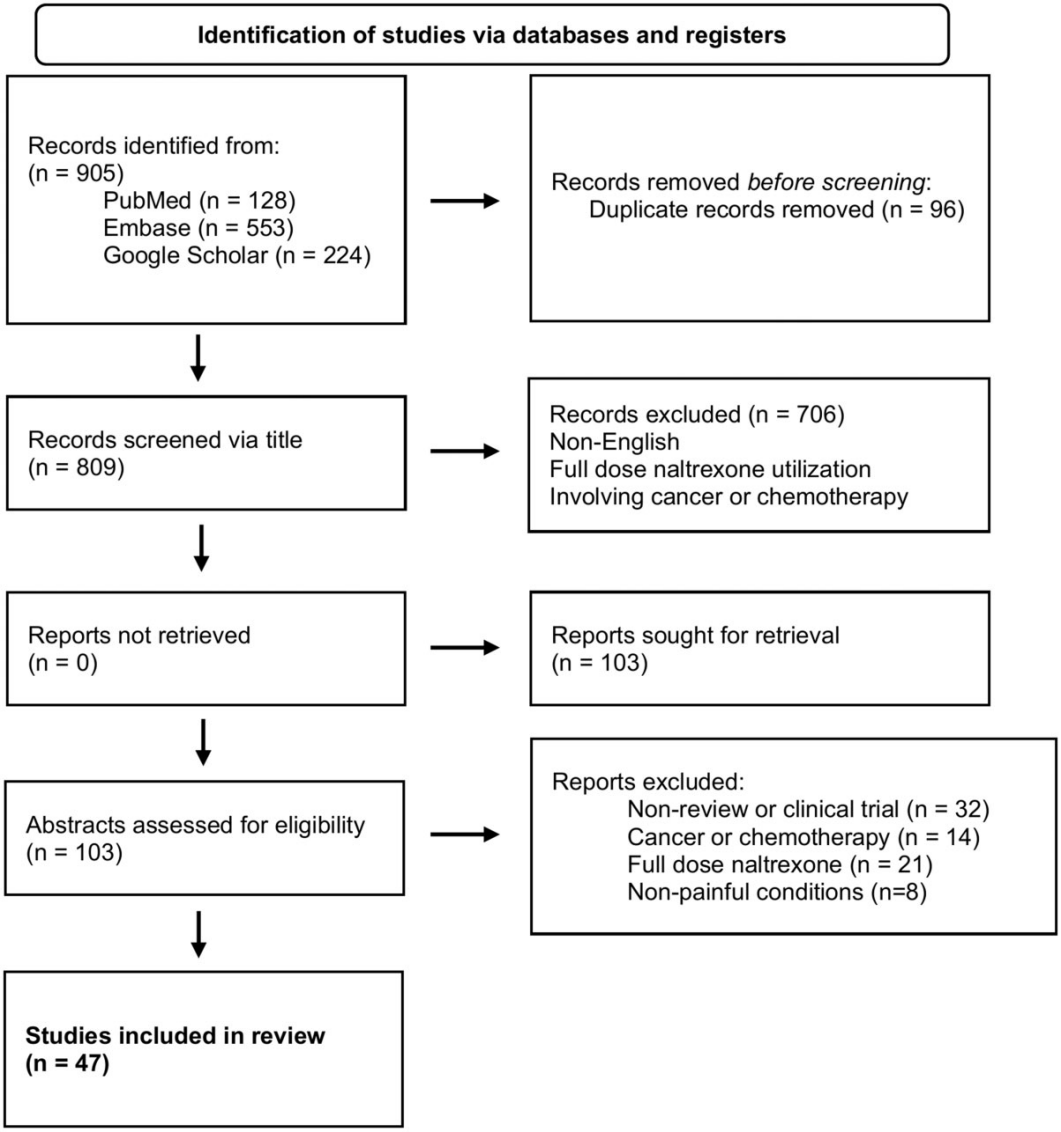
Flow chart methodology for the identification, screening, eligibility, and inclusion and exclusion process.

All search results were screened for eligibility and inclusion, starting with an initial title review, followed by an abstract and full-text review. To identify additional and missed pertinent studies after the initial review, the snowball strategy was used, in which all references and citations were reviewed. Publications not meeting the inclusion criteria were excluded from this review, and all duplicates were removed. Records meeting the inclusion criteria were further divided into categories by the specific condition being managed by LDN, as noted in Table 2. A total of 809 articles were initially screened via title, of which 706 were removed because of failure to meet the inclusion/exclusion criteria. Relevant study abstracts were reviewed, and 47 studies met the final criteria (Figure 1).

**Table 2.** Studies meeting inclusion criteria by condition treated

Condition	Total	Case reports or series	Cohorts	RCTs	Narrative reviews	Systematic reviews
CRPS	9	3	–	–	4	2
IBD	10	1	2	2	3	2
Fibromyalgia	18	2	3	1	10	2
Low back pain	4	1	–	–	2	1
Rheumatoid arthritis	1	–	1	–	–	–
Diabetic neuropathy	6	1	–	1	3	1

CRPS = complex regional pain syndrome; IBD = inflammatory bowel disease; RCT = randomized controlled trial.

Some systematic and narrative reviews might have included overlapping syndromes and were documented as such.

Variables were tabulated, including study design, number of human subjects, follow-up duration, clinical outcomes, and adverse effects (Table 3). For systematic and narrative review articles, articles were assessed for information as previously described, and relevant additional data were summarized and reported. If no new information was provided, the studies were noted in the counts only. The Scale for the Assessment of Narrative Review Articles (SANRA) criteria were used as a guide for this review to improve standardization.

**Table 3.** Compiled literature search results for LDN, including centralized condition, study design, outcomes, and adverse effects

Author, year	Type	*n*	Dosage	Outcomes	Adverse effects
**Complex regional pain syndrome**					
Chopra, 2013^19^	Case series	2	Patient #1: 4.5 mg Patient #2: 3 mg	Patient #1: 2 months NRS 10/10 → 5/10 Walking without cane Patient #2 3 months NRS 10/10 → 3/10 18 months Pain 100% resolved	None
Weinstock, 2016^20^	Case report	1	3 mg	16 months Pain 100% resolved	None
Sturn, 2016^21^	Case report	1	1.5 mg	4 weeks VAS 7/10 → 1/10	None
**Inflammatory bowel disease**					
Lie, 2018^22^	P-SC	47	4.5 mg	12 weeks Subjective clinical improvement: 74.5% Remission of symptoms: 25.5% Improvement in appearance of mucosa and epithelium noted by endoscopy	4 Vivid dreams 2 Drowsiness 1 Headache
Raknes, 2018^23^	Obs	256	Unspecified but <5 mg	Reduction in medication class by %: Anti-inflammatories –17 Immunosuppressants –29% Systemic corticosteroids –14% Intestinal corticosteroids –32% Aminosalicylates –17%	None
Smith, 2013^24^	P-RCT-CO	12 peds	Children over 10 and 45 kg, 4.5 mg Children under 10 or less than 45 kg, 0.1 mg/kg	16 weeks (LDN) 8 weeks (crossover group) CDAI: LDN vs baseline *P* = 0.005 LDN vs placebo *P* > 0.05 25% achieved remission in LDN group QoL in LDN group improved from baseline *P* = 0.035	Vivid dreams Nausea
Smith, 2011^25^	P-RCT-DB-SC	34 (16 control, 18 LDN)	4.5 mg	12 weeks CDAI score 88% (LDN) vs 40% (control) >70-point reduction (*P* = 0.009) Mucosal response 78% vs 28% (control) *P* = 0.008 Endoscopic remission 33% (LDN) vs 8% (control) *P* > 0.05 QoL *P* = 0.3 (favoring LDN)	Only LDN 2 Decreased appetite 2 Constipation Both 10 Insomnia 5 Unusual dreams 6 Headache 10 Abdominal pain 8 Nausea
Shannon, 2010^26^	Case report	1	4.5 mg	4 weeks Symptoms improved 3 months Endoscopic and biopsy results Crohn’s flare completely resolved	None
Ploesser, 2010^27^	R-Review	121 total *n* = 8 IBD (4 Crohn’s, 4 UC)	4.5 mg	16.8 weeks (average) 2/8 Markedly improved 1/8 Moderately improved 1/8 Mildly improved 4/8 Unchanged (though 2/4 were in remission before starting LDN)	74/121 AE 20/74 d/c LDN 2/2 AE Headaches (12%) Anxiety (16%) Insomnia (8.3%) Muscle pain (8.3%) Vivid dreams (5%) Abdominal pain (12%) Diarrhea (8.3%)
Smith, 2007^28^	P-SC	17	4.5 mg	16 weeks CDAI improved (*P* = 0.01) 67% achieved remission (*P* = 0.001) QoL significantly improved	5 Insomnia 2 Vivid dreams
**Fibromyalgia**					
Jackson, 2021^29^	P-SC	21	Started at 0.1 mg twice a day and by a week titrated up to 4.5 twice a day	7 weeks CPT doubled (*P* = 0.003) VAS 6/10 → 1/10 Subjectively improved fatigue, sleep, mood, work capacity	None
Rivera, 2019^30^	Case report	1	2.5 mg	4 days 242 MME → 0 MME VAS 9/10 → 5/10	None
Oaks, 2018^31^	Retrospective review	254	0.1 mg/day titrating up gradually to 4.5 mg/day	Baseline CPT 25.1 seconds 14 weeks CPT increased to 46.6 seconds (*P* = 0.008) 26 weeks CPT increased to 45.6 seconds (*P* = 0.03) 30 weeks CPT increased to 50.3 seconds (*P* = 0.03)	None
Parkitny, 2017^32^	P-SC-CO	8	4.5 mg (3.0 mg if patient had side effects)	10 weeks Reduction in cytokines associated with allodynia 15% reduction in pain 18% reduction in symptoms	None
Younger, 2013^33^	SC-RCT-DB-CO	31	4.5 mg	12 weeks Symptom reduction LDN 28.8% vs placebo 18% (*P* = 0.016) Successful response LDN 32% vs placebo 11% *P* = 0.05 Improvement in mood LDN > placebo (*P* = .039)	Headaches Vivid dreams more than placebo (*P* < .05)
Ramanathan, 2012^34^	Case report	1	1 mg for 2 nights, then 2 mg for 2 nights, then 4.5 mg	8 weeks VAS 7/10 → 3/10 Subjectively improved fatigue, sleep, mood, work capacity CPT 7 seconds → 50 seconds	Body ache and diarrhea—resolved after 2 months
Younger, 2009^35^	SC-DB-CO	10	4.5 mg daily	8 weeks Symptom reduction LDN 32.5% vs placebo 2.3% (*P* < .003) LDN > placebo for reduction in pain, fatigue, stress (*P* < .05) LDN > placebo for sleep, gastrointestinal symptoms, headaches, concentration, sadness (*P* = .05–.3)	2 Vivid dreams 1 Insomnia
**Low back pain**					
Ghai, 2014^36^	Case report	1	2 mg for 2 weeks, 4 mg for 4 weeks	2 weeks Pain reduced by 30%–40% 4 weeks VAS 10/10 → 3.5/10 MODQ 70% → 35.5% Medical leave → working full time 6 months (LDN d/c at 6 weeks) Ongoing significant pain relief	None
**Rheumatoid arthritis**					
Raknes, 2019^37^	Chart review	105	Unspecified, <5 mg	1 year All meds reduced by 13% (*P* = .003) Opioids reduced by 47% (*P* < .001)	None
**Painful diabetic neuropathy**					
Srinivasan, 2021^38^	RCT-DB-CO	67	2–4 mg LDN	6 weeks LDN VAS improvement *P* < .001 Amitriptyline VAS improvement *P* < .001 *P* value between groups .21	LDN (*n* = 8) Amitriptyline (*n* = 36) *P* < .001
Hota, 2016^39^	Case report	1	1, 2, and 4 mg for 2 weeks each	2 weeks VAS 9 → 0.5	Diarrhea

AE = adverse effects; CDAI = Crohn’s Disease Activity Index; CO = crossover; CPT = cold pressor test; d/c = discontinued; DB = double blind; LDN = low-dose naltrexone; MME = morphine milligram equivalents; MODQ = Modified Oswestry Disability Questionnaire; NRS = numeric rating scale; Obs = observational; P = prospective; QoL = quality of life; R = retrospective; RCT = randomized control trial; SC = single center; VAS = visual analog scale.

## Results

### Chronic regional pain syndrome

With regard to the use of LDN for the treatment of CRPS, 2 systematic reviews, 4 narrative reviews, and 3 case reports were identified. CRPS is a complex pain condition, encompassing both neuropathic and nociplastic pathophysiology, that includes a variety of pain and sympathetically mediated symptoms arising after peripheral injury not related to specific damage to the nervous system (CRPS type I, previously referred to as reflex sympathetic dystrophy) or arising from a specific peripheral nerve injury (CRPS type II, previously referred to as causalgia). The initial insult engages peripheral nervous system and CNS sensitization processes that have been shown to drive the autonomic and vasomotor symptoms and allodynia/hyperalgesia.^16^ In CRPS, CNS sensitization has been reported to depend on glial activation and a subsequent increase in proinflammatory cytokine release.^17^ As previously stated, the increased activation of microglial cells leads to a proinflammatory state, with secretion of pro-nociceptive neurotrophic factors resulting in enhanced excitatory and diminished inhibitory signaling within the CNS nociceptive networks, ultimately causing hyperalgesia and allodynia.^2^ Along these lines, postmortem immunohistochemical analysis of the spinal cord of a patient with CRPS has demonstrated upregulation of microglial TLR4 receptors, as well as overactivation of microglial cells within the CNS of patients with longstanding CRPS, specifically in the dorsal horn at the original level of injury.^18^

The first case series documented by Chopra et al.^19^ involved 2 patients with chronic (<3 years) right lower-extremity CRPS type I unresponsive to conventional treatment. In addition to significant numeric rating scale pain severity scores of 10 out of 10, the first patient also had lost the ability to ambulate unassisted. This patient was subsequently initiated on 4.5 mg LDN daily. At 2 months after LDN initiation, the patient’s pain severity improved from a numeric rating scale score of 10 out of 10 to a score of 5 out of 10, and the patient had regained the ability to ambulate without assistive devices. The second patient in this case series involved a 12-year-old patient with Ehlers-Danlos syndrome who developed an insidious onset of right lower-extremity CRPS type I refractory to traditional conservative management. Three months after initiation of 3 mg LDN, her pain severity improved from a numeric rating scale score of 10 out of 10 to a score of 3 out of 10, and by 18 months of treatment, her pain had completely resolved (0 out of 10).^19^

In the second case report, Weinstock et al.^20^ reported the use of LDN in a 56-year-old female with right lower-extremity CRPS type I that reportedly emerged after a cardiac catheterization procedure. Her symptoms had been present for 8 years and had been refractory to conservative treatment. The authors reported that by 16 months after initiation of 3 mg LDN daily, her pain had completely resolved.^20^

Sturn et al.^21^ published the third case report, which involved a 17-year-old girl with left lower-extremity CRPS type I of unknown etiology that had persisted for at least 2 years and was refractory to conservative management, including clonidine, nortriptyline, and gabapentin. In addition, she was unable to wear regular shoes. She was subsequently started on 1.5 mg LDN, and her symptoms improved from a visual analog scale (VAS) score of 7 out of 10 to a score of 1 out of 10 within 4 weeks, and she was also able to wear regular shoes.^21^

In the aforementioned case reports and series, none reported adverse effects while using LDN in these 4 patients with CRPS (Table 3).

### Inflammatory bowel disease

On the topic of the use of LDN to treat pain and symptoms related to inflammatory bowel disease (IBD), 2 systematic and 3 narrative reviews were analyzed, along with 2 RCTs, 2 prospective cohorts, and 1 case report. Two of the included studies (Lie et al. and Ploesser et al.) evaluated IBD (including both ulcerative colitis [UC] and Crohn’s disease), whereas the remaining studies focused exclusively on Crohn’s. For the evaluation of disease severity in active Crohn’s, the Crohn’s Disease Activity Index (CDAI) score evaluates symptoms (such as abdominal pain), weight, laboratory values, and extra-intestinal findings (such as arthritis and dermatological complications). A moderate to severe flare would score between 220 and 450, and remission is suggested at scores of <150.^40^ In addition, the Crohn’s Disease Endoscopic Index of Severity score (CDEIS), which focuses on the extent of involvement as visualized on endoscopy, can also be used. Scores range from 0 to 44, with higher scores reflecting more severe disease burden, though this metric does not include pain reports specifically.^41^

IBD is an umbrella term and includes both Crohn’s and UC. Crohn’s and UC share similar pathophysiology and symptoms, including abdominal pain, diarrhea, and hematochezia, but they differ in location and depth of inflammation within the enteric system.^42^

The specific etiology of IBD remains unknown, but it is suspected to result from a combination of environmental and genetic risk factors. The pathogenesis, on the other hand, has been extensively studied and involves a dysregulation of the immune response toward host mucosal antigens or bacteria.^28^^,^^43^ Pain is a very common symptom in IBD and is often the presenting sign in up to 70% of patients. Initially, this is due to inflammation and mucosal irritation, but a high percentage of these patients (30%–50%) will continue to have pain even when clinically in remission.^44^ It is suspected that peripheral to central sensitization mechanisms are the cause of this persistent pain, as shown in animal studies in which hypersensitivity of mechanoreceptors and nocioreceptors was seen after the induction of colitis.^45^^,^^46^ In addition to this centralized pain, it has been described that while in active disease, the gastrointestinal immune system becomes dysregulated, which leads to an upregulation of µ-opioid receptors on CD4+ and CD8+ cells.^47^ Intermittent blockade of these upregulator receptors has been shown to improve pain, diarrhea, and anorexia.^48^^,^^49^ This leads to the overall hypothesis that LDN could act to improve symptoms in both active IBD, through the µ-opioid receptor, and in centralized pain, via glial cell modulation.

The first prospective study, conducted by Smith et al., involved 17 subjects with chronic refractory active Crohn’s disease with symptoms including abdominal pain, hematochezia, malabsorption, and diarrhea. Eligible patients had histological and endoscopic evidence of active Crohn’s disease on the CDEIS.^28^ Sixteen weeks after patients had started a regimen of 4.5 mg daily LDN, there was a significant reduction in CDAI scores (*P* = .01), with 67% of patients achieving remission (*P* = .001). Health-related quality of life was found to be significantly improved compared with baseline. Adverse effects were minimal but were reported in a few patients; they included vivid dreams (*n* = 2) and insomnia (*n* = 5).

Smith et al. also conducted the first prospective double-blind single-center RCT study evaluating the effects of LDN in patients with Crohn’s disease.^25^ The study included 34 patients randomized to either placebo (*n* = 16) or 4.5 mg LDN daily (*n* = 18). At 12 weeks, 88% of patients randomized to LDN had a 70-point decline in CDAI scores, compared with 40% of the placebo group (*P* = .009). Only 30% of the LDN group achieved remission on the basis of CDAI scores, but the authors noted that the cohort’s baseline CDAI scores were very high, and remission is difficult in patients with high baseline CDAI scores. In addition to the CDAI outcome measures, CDEIS scores improved in 78% of patients in the LDN group, compared with 28% in the placebo group (*P* = .008), while complete remission was noted in 33% of the subjects who took LDN and 8% of the placebo group (no significant difference, *P* > .05). Quality of life was clinically improved in the patients who took LDN compared with the placebo group, though this did not reach statistical significance (*P* = .3). Unique adverse effects reported in the LDN group included loss of appetite (*n* = 2) and constipation (*n* = 2), though these were not significantly different from placebo. Both groups experienced insomnia (*n* = 10), unusual dreams (*n* = 5), headache (*n* = 6), abdominal pain (*n* = 10), and nausea (*n* = 8).

In 2013, Smith et al. conducted a follow-up RCT with a crossover design, evaluating LDN in 12 children with moderate to severe Crohn’s disease.^24^ Each treatment arm contained 6 participants. One group received LDN for 16 weeks, and the other received placebo for 8 weeks and then crossed over and received LDN for 8 weeks. LDN doses were weight based but were roughly 4.5 mg for most participants. At 8 weeks, CDAI scores significantly decreased compared with pretreatment scores (*P* = .005) for the LDN group. Because of the small sample size of the cohort, when LDN was compared with the effect of the placebo group, the difference between the CDAI scores did not reach statistical significance. Remission based on CDAI scores was achieved in only 25% of patients who took LDN, but quality of life significantly improved (*P* = .035) in patients who took LDN compared with placebo groups. Adverse events that were recorded included vivid dreams and nausea when patients were taking LDN as compared with placebo.

Shannon et al. wrote on a case report of a 14-year-old female patient with refractory Crohn’s disease showing erosions in the duodenum and biopsy consistent with duodenitis.^26^ She was initially trialed on prednisone and azathioprine, but this resulted in severe myalgias and stiffness that required a wheelchair. She was placed on 4.5 mg LDN daily and had significant improvement in her symptoms after 4 weeks. In addition, endoscopic evaluation at 3 months after LDN initiation showed complete mucosal healing with normal biopsies. It is unclear how these improvements were measured in the case report. No adverse events were reported.

Raknes et al. conducted an observational study evaluating LDN’s effect on the use of other medication for Crohn’s disease.^23^ In 256 identified patients who persistently took LDN (denoted as picking up >4 prescriptions over an undefined period of time), they found overall reductions in various prescriptions, including anti-inflammatories (–17%), immunosuppressants (–29%), systemic corticosteroids (–14%), intestinal corticosteroids (–32%), and aminosalicylates (–17%). No adverse effects were reported.

The last 2 studies are the only ones that also included UC in their patient populations. Lie et al. conducted a prospective cohort study involving 47 patients with refractory IBD.^22^ Participants were given 4.5 mg LDN daily and then followed up for 12 weeks. At 12 weeks, the authors found subjective clinical improvement in 74.5% of patients (based on self-assessments) and remission in 25.5% (though it is unclear how remission was defined). Mucosal and epithelial barrier evaluations improved, according to a study-specific scale for endoscopic evaluation. Adverse effects included vivid dreams, drowsiness, and headache (*n* = 4, 2, and 1, respectively).

Ploesser et al. conducted a retrospective chart review involving 121 subjects from a single gastroenterologist’s clinic who had various diagnoses, including irritable bowel syndrome, IBD, and chronic idiopathic constipation, and were prescribed LDN.^27^ As this was mainly a safety study, patients with and without refractory IBD were included. Patients with IBD were given 4.5 mg daily. Although a minority of the participants (*n* = 8) had IBD (Crohn’s *n* = 4, UC *n* = 4), the overall side effect profile of the study was relatively low, with 74 of the 121 subjects reporting mostly transient adverse effects, including headaches, anxiety, insomnia, muscle pain, vivid dreams, abdominal pain, and diarrhea, which resolved with extended use. However, 20 of the 74 discontinued LDN because of side effects. Of the patients with IBD, the average duration of treatment was 16.8 weeks. Of the 8 subjects with IBD, 2 had marked improvement, and 4 of the 8 subjects were unchanged (based on a patient-reported study-specific survey). It should be noted, however, that 2 of the 4 subjects with unchanged pain were in remission before they started LDN.

### Fibromyalgia

With regard to the use of LDN for FM, 2 systematic reviews, 9 narrative reviews, 1 RCT, 3 prospective cohorts, 1 chart review, and 2 case reports have been published.

FM is a chronic pain condition estimated to affect 1% to 6% of the general population and characterized by widespread musculoskeletal pain, hyperalgesia, and allodynia.^50^^,^^51^ The pathophysiology of FM is complex and multifactorial, but it has been described as the prototypical centralized pain syndrome in which the majority of the pain experienced is nociplastic. Patients with FM often have concurrent disabling somatic symptoms, including chronic fatigue, concentration and memory issues, anxiety, depression, headaches, irritable bowel syndrome, and interstitial cystitis.^51^^,^^52^ Central sensitization is thought to be a large component of the pathophysiology underlying FM and most other chronic overlapping pain syndromes.^51^ Chronic neuroinflammation with pro-nociceptive cytokine profiles due to microglial activation has been described in patients with FM,^53^ leading to the hypothesis that compounds that regulate microglial activation, like LDN, could have a positive impact on FM symptoms.^11^

Younger et al. were the first to report a positive impact of LDN on pain and pain-related symptoms in patients with refractory FM.^35^ They conducted a single-blind crossover pilot trial in 10 women with FM. Participants self-reported their daily symptoms on study-specific questionnaires nightly for the duration of the study. The primary outcomes included a single symptom severity (0–100) question, with secondary endpoints assessing average daily pain, highest pain, fatigue, sadness, stress, sleep quality, ability to think and remember, gastrointestinal symptoms, and headaches. After the patients had taken 4.5 mg LDN daily for 8 weeks, Younger et al. observed a 32.5% reduction in overall FM symptoms for the LDN group, compared with 2.3% for the placebo group (*P* < .003), as well as significant reductions in daily pain, fatigue, and stress (*P* < .05) compared with placebo. Only 2 subjects reported adverse events in the form of vivid dreams, and 1 reported insomnia.

Subsequently, Younger completed a larger follow-up trial, a double-blind crossover RCT, in which 31 women with FM were evaluated.^33^ This trial consisted of a 4-week placebo and a 12-week LDN (4.5 mg daily) period with a crossover after each treatment arm. In agreement with the pilot study, 12 weeks of LDN reduced symptoms compared with placebo (28.8% vs 18%, *P* = .016), and the LDN group saw statistically significant improvement in mood (*P* = .039) compared with placebo. Successful response to treatment was defined as a significant reduction in self-reported pain severity and fatigue (defined as a reduction of >30% for each category). This was reported in 32% of the LDN treatment arm vs 11% of the placebo group (*P* = .05). In this study, the side effects of LDN included more frequent headaches and vivid dreams compared with placebo (*P* < .05).

Taking this further, in a single-blind crossover study in 8 women with FM, Parkitny et al. reported that FM was associated with increased release of various interleukins, proinflammatory cytokines, and growth factors.^32^ Participants were to have their blood drawn twice weekly to evaluate for circulating cytokine levels. In addition, daily pain and general symptom severity scores (0–100) were captured. After 10 weeks of 4.5 mg LDN daily, there was a 15% reduction in FM-related pain severity and an 18% reduction in other symptoms, which corresponded to decreased circulating levels of cytokines known to promote allodynia and hyperalgesia.^54^^,^^55^

Separately, Jackson et al. reported on a prospective cohort study involving 21 patients with FM. After 7 weeks of LDN treatment at 4.5 mg twice a day, their cold pressor test scores doubled (*P* = .003), their VAS scores improved from 6 out of 10 to 1 out of 10, and self-reported improvements were seen in fatigue, sleep, mood, and work capacity.^29^

Ramanathan et al. presented a case involving a 37-year-old male with diffuse pain, fatigue, weakness, and insomnia who was subsequently diagnosed with FM. Conservative management failed.^34^ Given the patient’s profile of refractory pain, 4.5 mg daily LDN was initiated for 2 weeks and then discontinued for another 2 weeks (because of a misunderstanding in the titration directions) before it was reinitiated for 14 weeks. By 8 weeks after reinitiation of the LDN, multiple modalities of pain were reduced. Specifically, the patient’s VAS score decreased from 7 out of 10 to 3 out of 10, and his duration of tolerance in the cold pressor test (in which subjects submerge their hands in ice water) improved from 7 to 50 seconds, which indicates a dramatic increase in objective pain tolerance. This resembled reference durations for normal healthy subjects. Additionally, he reported subjective improvements in fatigue, sleep, work capacity, and mood.

Rivera et al. presented a second case report involving a 45-year-old female with co-occurring chronic pelvic pain, FM, and interstitial cystitis.^30^ After opioid therapy with oxycodone doses of up to 242 morphine equivalents daily failed, the patient was started on 2.5 mg/day of LDN. Four days after initiation of LDN, she was weaned off her oral opiates, and after a follow-up period of unknown duration, she had a reduction in her pain severity, from a VAS score of 9 to a score of 5.

Oaks et al. performed a retrospective review involving 254 patients with FM or opioid-induced hyperalgesia who were started on LDN.^31^ After initiation of 4.5 mg LDN, they found statistically significant improvements in cold pressor test scores (*P* = .008) for both opioid users and patients with FM at 14, 26, and 30 weeks.

### Low back pain

We found 1 systematic review analysis, 2 narrative reviews, and 1 case report discussing LDN and lower back pain.

The pathophysiology behind how LDN could be a therapeutic option for chronic low back pain is likely related to its effect on central sensitization and neural glial modulation, as central sensitization has been identified as being highly relevant in patients with chronic low back pain.^56^^,^^57^ Ghai et al. described a case report involving a 35-year-old male with nonspecific axial low back pain that had been present for >2 years.^36^ His average pain severity VAS score was 10 out of 10, and his Modified Oswestry Disability Questionnaire (MODQ) score was 70%. After multiple conservative strategies, including medications and interventional pain injections, had failed, the patient was started on 2 mg LDN and uptitrated to 4 mg daily. At 2 weeks after LDN initiation, his pain severity was reduced by 30% to 40%. At 4 weeks after LDN initiation, his pain severity VAS score decreased to 3.5, and his MODQ score reduced to 35.5%. In addition, after LDN treatment, he was able to resume a part-time job. LDN was discontinued after a total of 6 weeks of treatment. At 6 months’ follow-up, he reported minimal pain and was working full time. No adverse events were reported.

### Rheumatoid arthritis

We found 1 retrospective study discussing rheumatoid arthritis and the use of LDN. Rheumatoid arthritis is a well-studied autoimmune disorder that results in chronic inflammation in multiple joints and a variety of other systemic manifestations. Rheumatoid arthritis is typically a peripheral joint disease, but, much like the other disease processes mentioned in the present review, continued inflammation and irritation of nociceptive receptors can lead to the centralization of pain over time.^13^ To this end, Raknes et al. conducted a retrospective review of patients with rheumatoid arthritis treated with LDN for 2 years at doses of <5 mg.^37^ A total of 180 patients were included in the persistent LDN usage group (defined as having filled >4 prescriptions for LDN), as opposed to patients who filled the prescription only once or twice. Medication amounts were tabulated on the basis of the number of prescriptions filled during that time frame. After 1 year, the persistent LDN group had reduced daily doses of all other medications by 13% (*P* = .003). These included nonsteroidal anti-inflammatory drugs, disease-modifying antirheumatic drugs, tumor necrosis factor alpha inhibitors, corticosteroids, and opioids. In addition, a 47% (*P* < .001) reduction in opioids was seen.

### Diabetic neuropathy

We found 1 systematic and 3 narrative reviews along with 1 RCT and 1 case report discussing LDN and diabetic neuropathy. It has been proposed that the pathophysiology of how diabetic neuropathy causes centralized pain involves the sensitization of primary afferents and dorsal horn neurons.^58^ It is believed that diabetes increases glutamate release and upregulates *N*-methyl-d-aspartate receptor expression, both of which enhance the excitation of neurons, specifically in lamina II.^59^ A second proposed mechanism is the downregulation of GABA_B_ receptors seen in diabetes, which has a close tie to the impaired inhibition of *N*-methyl-d-aspartate receptors.^60^ Microglial cells have been shown to play an additional role in the centralization of pain in patients with diabetes.^58^

Srinivasan et al. conducted an RCT comparing the efficacy of 2 mg LDN and 10 mg amitriptyline for painful diabetic peripheral neuropathy.^38^ A total of 67 participants were randomized to either trial arm and followed up every 2 weeks for 6 weeks. If their VAS score did not improve by >20%, the doses were increased to a maximum of 4 mg LDN and 50 mg amitriptyline. After 6 weeks and a 2-week washout period, the groups were crossed over, and the trial was repeated. The authors found similar efficacy for both medications, but they did note a better safety profile with LDN than with amitriptyline. The total number of adverse events for LDN was significantly lower than that for amitriptyline. The notable adverse reactions that occurred significantly less often for LDN were insomnia, postural hypotension, and dry mouth. Other adverse events, such as diarrhea, nausea, and headache, were reported nonsignificantly in both groups.

Hota et al. presented the case reports on LDN and diabetic neuropathy.^39^ The authors reported a patient who initiated LDN with refractory diabetic neuropathy and hyperalgesia of both feet. He started at 1 mg/day for 2 weeks, then 2 mg/day for 2 weeks, and then finally was titrated to 4 mg/day. His pain severity VAS score improved from 9 to 0.5 after 2 weeks of LDN treatment, and he continued to show benefit while on LDN treatment at a 2-year follow-up. The patient had only mild diarrhea for the first few days after LDN initiation.

## Discussion

LDN is a relatively novel yet encouraging method for managing chronic centralized pain stemming from conditions such as FM, IBD, and CRPS. Development of new management strategies for these specific conditions is crucial, given the complex and unique challenges they pose to medical professionals. Although studies are limited mostly to case reports and narrative reviews, some RCTs have been completed. Promising results affecting pain and hyperalgesia have been shown in the RCTs and clinical trials, specifically for IBD and FM.^24^^,^^25^^,^^33^ These clinically and statistically significant reductions in pain and hyperalgesia were not only found but then reproduced in subsequent cohort studies.^22^^,^^28^^,^^29^^,^^32^^,^^35^

LDN’s impact on centralized pain originates from the compound’s innate ability to modulate the CNS glial cells via TLR4.^61^ This ultimately breaks the glial cell activation cycle and reduces cytokine release.^7^ The pain conditions reviewed in the present article stem from pathophysiological changes within the CNS and nervous glial cells, further supporting the utility of LDN in managing these patients.^62^

In addition to improved pain, there were multiple reports of improved function,^19^^,^^36^ sleep,^33^^,^^34^^,^^63^ mood,^33^^,^^34^ and quality of life^24^^,^^25^ with the use of LDN. This can likely be attributed to the typical concurrent symptoms associated with the conditions.^64^^,^^65^ Although LDN is primarily a microglial cell modulator, the improvements seen in function, sleep, quality of life, and mood allude to the tight relationship between those and pain.^65^^,^^66^

On the basis of the present review, LDN is generally well tolerated by most patients with chronic pain. Adverse effects were commonly reported but were minimal to mild at worst, with the most common being vivid dreams, diarrhea, and headaches.^67^^,^^68^ Only rarely did adverse effects require patients to terminate treatment.^19^ More commonly in this situation was that as the dose was increased or the trial persisted, the patient’s side effects dissipated with time.^25^^,^^27^ Additionally, in the review conducted by Srinivasan et al., LDN was found to have efficacy similar to that of amitriptyline for diabetic peripheral neuropathy but was associated with a significantly lower adverse effect profile.

Throughout the investigated trials, there was significant variation in the timing of follow-up assessments, with study designs ranging from a day to a year, with the average follow-up time roughly 12 weeks.^37^^,^^63^ During these follow-ups, patients had variable responses to LDN, with some showing improvement quickly and some only after more time had passed.^21^^,^^37^^,^^39^ Despite the inconsistent timing of improvements, there does appear to be an increase in efficacy over time, with a large subset of patient seeing some benefit.^20^^,^^26^^,^^36^^,^^63^

Dosing varied among the studies, ranging from 0.2 mg to 9 mg daily, but 4.5 mg daily was the most used dosing regimen. Currently, it is well published that 4.5 mg daily is a feasible target dose for LDN. This is likely because of LDN’s paradoxical effect, in which doses up to 4.5 mg daily appear to enhance opioid signaling, whereas at higher doses this effect disappears and opioid antagonism begins.^57^^,^^69^ Nevertheless, efficacy was seen with doses as low as 1 mg^39^ and as high as 9 mg.^29^ It should be noted that under the titration scheme used in many of these published reports of patients taking LDN, patients were usually started on a lower dose and were titrated accordingly on the basis of their pain and side effects, usually at initial follow-up assessment. Thus, it is unclear whether the improvements seen at subsequent visits are time or dose dependent, which is a limitation of the rigor of the prior research. Further studies could be conducted focusing on when the patient first noted changes in symptoms, in addition to trialing various doses without adjustments.

Multiple reviews have been published that have alluded to the direct benefit of LDN on chronic pain, quality of life, sleep, and anxiety.^67^^,^^68^ However, there are also several secondary effects of LDN that are less studied but mentioned in the literature.^70^ One of the important synergies associated with LDN is that LDN has been shown to improve a patient’s response to opioid medications.^71^^,^^72^ Preclinical mouse studies have reported increased anti-nociceptive potency of morphine in mice that received LDN as compared with controls.^73^ In contrast, there was no significant difference in trauma patients who received concurrent morphine and LDN, as shown in the study by Farahmand’s et al.^74^ Although this is interesting, it is often reported that patients in whom LDN is being considered should not be taking opioid agonists, and in general, opioids are not recommended for the treatment of chronic pain, especially FM and other centralized pain syndromes, because of the prevalence of and concern about opioid-induced hyperalgesia. It is clear that more controlled studies are required to support or refute these synergistic effects. However, if found reputable, these secondary attributes could have the potential to improve utility, acceptance, and funding. Given the positive and encouraging findings from the body of literature presented in this review, along with the ongoing off-label use of LDN, further high-quality studies would be beneficial to improve recognition, standardization, and expanded usage.

## Conclusion

LDN is a novel therapeutic treatment option for patients with centralized chronic pain. Numerous small case reports and a few higher-quality trials support the use of LDN in chronic pain. Despite this, LDN is still used off label for many chronic pain conditions. It is imperative that higher-quality, well-designed, and controlled studies be conducted to expand and potentiate the application of LDN and its utility profile, as well as help with standardization of treatment regimens. Even though such studies are much needed, LDN still offers promising results in the current clinical management of refractory symptoms in patients with these chronic centralized pain conditions.
